# Probing Antigen-Antibody Interaction Using Fluorescence Coupled Capillary Electrophoresis

**DOI:** 10.3390/ijms140919146

**Published:** 2013-09-17

**Authors:** Jianhao Wang, Lin Qiu, Cheli Wang, Yue Zhang, Jingyan Li, Jiang Xia, Pengju Jiang

**Affiliations:** 1 School of Pharmaceutical Engineering and Life Science, Changzhou University, Changzhou 213164, China; E-Mails: minuswan@gmail.com (J.W.); linqiupjj@gmail.com (L.Q.); clwang@cczu.edu.cn (C.W.); zyjs@cczu.edu.cn (Y.Z.); jingyan.leee@gmail.com (J.L.); 2 Department of Chemistry, the Chinese University of Hong Kong, Shatin, Hong Kong

**Keywords:** QDs, FRET, immunocomplex, capillary electrophoresis

## Abstract

In this report, the use of fluorescence detection coupled capillary electrophoresis (CE-FL) allowed us to fully characterize the antigen-antibody interaction. CE-FL allowed separation of unbound quantum dots (QDs) and ligand bound QDs and also revealed an ordered assembly of biomolecules on QDs. Further, we observed FRET from QDs donor to DyLight acceptor, which were covalently conjugated with human IgG and goat anti-human IgG, respectively. The immunocomplex was formed and the mutual affinity of the antigen and antibody brought QDs and DyLight close enough to allow FRET to occur. This novel CE-based technique can be easily extended to other FRET systems based on QDs and may have potential application in the detection of antibodies.

## 1. Introduction

Quantum dots (QDs) are finding increasingly wide uses in biolabeling as they possess remarkable characteristics over organic fluorophores, such as high quantum yield, large Stokes shift, broad absorption spectra, low levels of photobleaching, long fluorescent lifetimes and size-tunable photoluminescent emissions [1–7]. To maintain their desirable fluorescent properties in aqueous biological fluidics, surface functionalization of QDs is a crucial step. Many strategies have now been developed to conjugate QDs with biomolecules. Among them, covalent conjugation is the most promiscuous method for QDs surface functionalization, including amide bond formation between carboxylic acids and amines [8–12], thiol-maleimide coujugation [13–15], click chemistry conjugation [16], halotag conjugation [17] and others.

FRET technology provides a fast, sensitive and simple way of dynamically monitoring life process by its nano-scale study of molecular structure and biological function. It plays an important role in nucleic acid detection [18], protein structure, function and its interaction [19], immune analysis [20], *etc*., and has become an important method in biomedical research. QD-based FRET biosensors have been widely used in immunoassay [21], biomedical sensor [22,23] and intermolecular binding assay [24,25].

In this report, QDs were covalently coupled to Protein A by activating agents. We demonstrated stoichiometry of the self-assembly between Protein A and QDs, and a substantially formation of QD-IgG assembly using CE-FL. The immunocomplex was then formed by adding DyLight-labeled Goat anti-human IgG, the antigen and antibody were close enough to allow FRET to occur. The efficient separation of immunocomplex from free donor and acceptor was achieved, which reduced the analysis uncertainty. This novel CE-based technique can be easily extended to other FRET systems based on QDs and may have potential application in the detection of antibodies.

## 2. Results and Discussion

Most analytical and physiochemical methods that are widely applied to antigen-antibody interaction studies, such as surface plasmon resonance (SPR) [26], enzyme-linked immunosorbent assay (ELISA) [27], high perfomance size exclusion chromatography (HPSEC) [28] and others. Especially in recent, Zhao *et al*. reported a simple but efficient electrochemical method to probe into the interaction between β-amyloid peptides and bilayer lipid membrane for revealing the toxic mechanism of Alzheimer’s disease [29]. This method might provide a convenient and powerful approach for in vitro studies of diseases.

There are mainly two strategies used to combine QDs with biomolecules. An alternative method of combination involves electrostatic attraction. This method is easier to operate, but not sufficiently stable. Another covalent method uses coupling agents to conjugate QDs to biomolecules, which is very stable by modification of QDs’ surface and performs certain advantages in the specific marking. Therefore, the coupling agents EDC and NHS were used to conjugate QDs and biomolecules.

**Figure 1:**
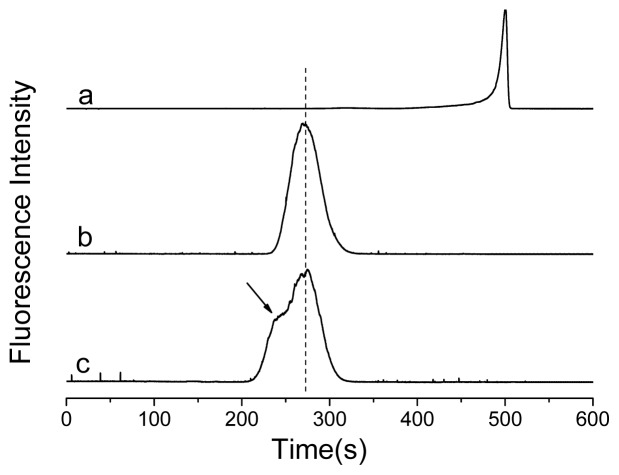
Electropherograms of quantum dots (QDs)-IgG conjugation with detection in 612 nm channel. (**a**), QDs alone; (**b**), QDs-Protein A; (**c**), QDs-IgG. (λ_ex_ = 420 nm).

SpeA and QDs mixtures were first chromatographed by CE-FL. CE-FL has been shown to be an effective method to detect QDs-protein interaction, which reveals subtle changes in the structure and composition of the surface bound ligands on QDs [21,30]. CE-FL can provide far more detailed information on QDs-protein assembly than ensemble fluorescence measurement [21]. Comparing with gel electrophoresis which is also used for QDs-protein assembly studies [31], CE-FL features faster separation, high reproducibility and higher maneuverability. QDs-protein assemblies with different stoichiometry can be separated based on mobility. Figure 1 shows the electropherograms of mixing Protein A with QDs. The electropherogram of the maximal emission wavelength of QDs, 612 nm in each electrophoretic run were extracted. CE could efficiently separate the bound and unbound species.

In order to choose the optimal ratio of QDs to Protein A, the conjugation of QDs and Protein A was detected by CE-FL. QDs showed a strong peak at 490 s (Figure 1, curve a), while for the conjugates (Figure 1, curve b), indicated by a stable species of QDs-Protein A in CE-FL with migration time of 270 s, significantly different from un-displaced QDs. By the location of the emission peak, this peak was known to be caused by the QDs-Protein A. After the conjugation of Protein A and QDs, the surface charge changed and the fluorescence peak moved forward. This implies an ordered assembly of Protein A on the surface of QDs instead of random binding. More informative QDs-IgG assembly analysis comes from CE studies. After adding human IgG, a new shoulderpeak was found at 240 s (Figure 1, curve c), it was deduced that this new shoulderpeak was caused by the formation of QDs-IgG.

**Figure 2:**
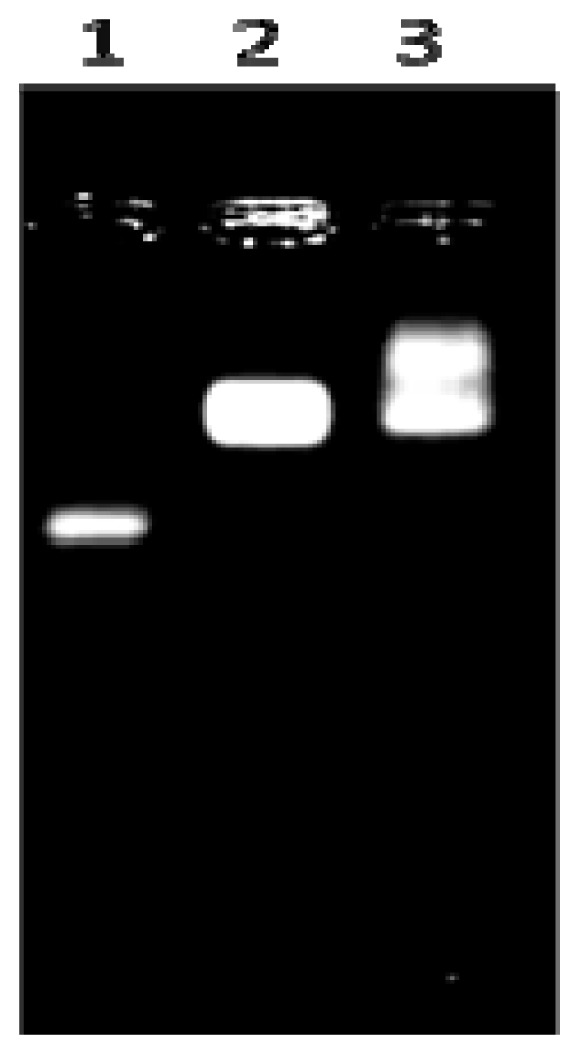
Electropherograms of QDs-IgG complexes in agarose gel. Line 1, QDs alone; Line 2, QDs-Protein A; Line 3, QDs-IgG.

We aimed to further prove that the shoulderpeak was caused by QDs-IgG. When we analyzed them by agarose electrophoresis, we observed a striking ladder of QD mobility (Figure 2) of both QDs-Protein A and QD-IgG. Therefore, it was proved that QDs-IgG formed successfully.

**Figure 3:**
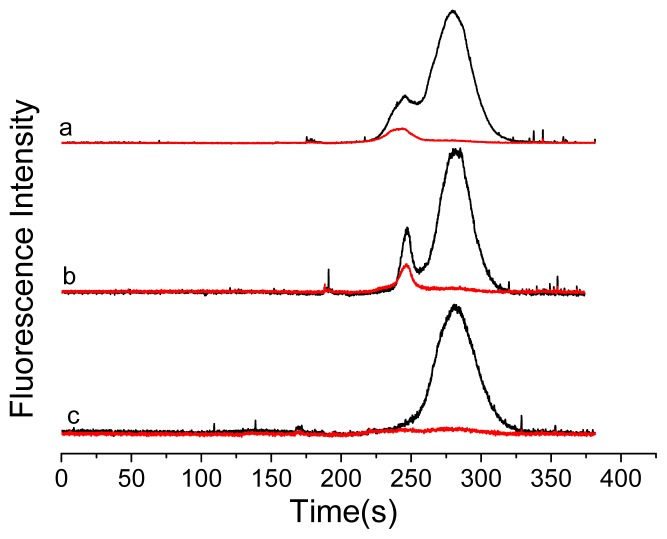
Electropherograms of QD-immunocomplex with detection in two different channels (black: 612 nm for QDs; red: 670 nm for DyLight). (**a**) 1 h immune reaction mixture; (**b**) 2 h immune reaction mixture; (**c**) 2 h immune reaction mixture after adding excess protein A for 1 h.

**Scheme 1:**
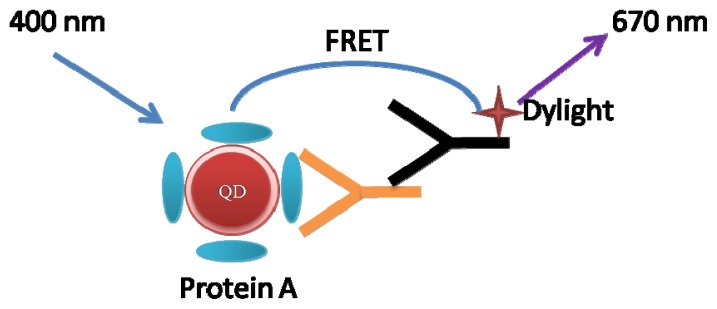
Schematic illustration of the FRET between QDs and Dylight.

These experiments above confirmed that IgG and QDs were effectively conjugated. To study the FRET process between fluorophores, DyLight labeled goat anti-human IgG was added to the conjugates (Scheme 1). The antigen-antibody reactions shortened the distance between QDs and Dylight. Experimental results (Figure 3) showed that after adding antibody labeled with DyLight, the fluorescence could be detected at 612 nm (QDs) and 670 nm (DyLight).

**Figure 4:**
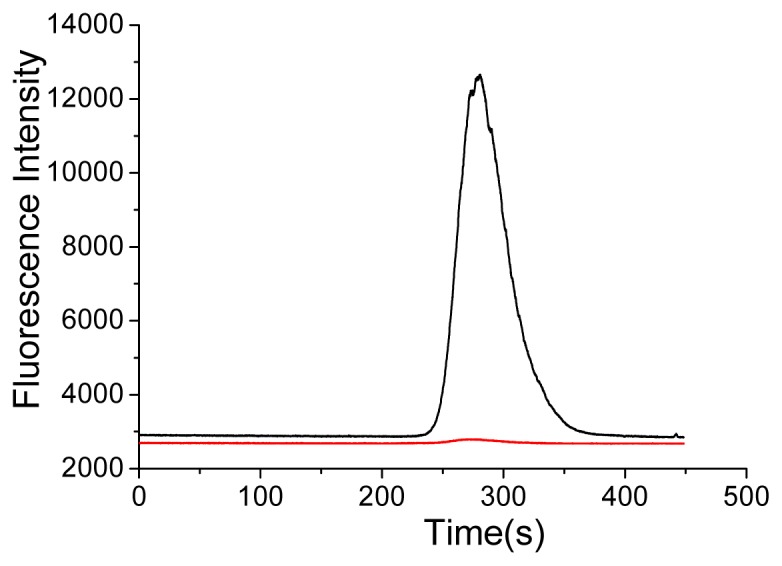
Electropherograms of the mixture of QDs, human IgG and DyLight labeled goat anti-human IgG with detection in two different channels (black: 612 nm for QDs; red: 670 nm for DyLight).

One hour later, the FRET signals with a new peak at 245 s were found at both 612 nm and 670 nm channels. This is because the FRET signals are decided by the amount of immunocomplex formed. At the beginning, the amount of immunocomplex was formed and increased gradually. And two hours later, it was found that the FRET signals increased and reached stability ever since. As a comparison, QDs and human IgG were mixed directly in the absence of Protein A. From Figure 4 it could be seen that only QDs signals were found after adding DyLight labeled antibody. In other words, QD-immunocomplex was not formed, which indicated that the FRET process could not happen in the absence of Protein A.

To further investigate the process between DyLight labeled antibody and QDs labeled antigen, the QD-immunocomplex was studied with adding excess Protein A to destroy the FRET system. It was found that the FRET signals disappeared after adding Protein A (Figure 3, curve c). This is because the pure FRET signals are decided by the amount of immunocomplex formed. Thus, it was further confirmed the FRET process did happened between QDs labeled antigen and DyLight labeled antibody. Therefore, we show that CE-FL can resolve antigen-antibody binding events owing to its superior resolution and the ability to simultaneously monitor multiple emission channels. This new proposed method can also be used to study the interaction between the species related to cancers. This method also allowed us to monitor the competition of different cancer related antigen with antibody.

## 3. Experimental Section

### 3.1. Materials and Instruments

Protein A, Human IgG and Goat Anti-Human IgG (DyLight649 Conjugated) were purchased from BeiJing Cowin Biotech Co. Ltd. (Cowin Biotech, Beijing, China). Glutathione (GSH) was purchased from Adamas-Beta Co. Ltd. (Adamas-Beta, Shanghai, China). All other chemicals and materials were of analytical grade. Ultrapure water (≥18.2 MΩ) purified by Milli-Q system (Millipore, Bedford, MA, USA) was used for preparation of all solutions. The electrophoresis buffers were filtered through a 0.22 μm filter before use.

Capillary electrophoresis analyses with fluorescence detection were carried out on a home-built system, consisting of a high voltage supply (0–30 kV) (Shanghai Nuclear Research Institute, Shanghai, China), a fused-silica capillary with an inner diameter (ID) of 75 μm (Yongnian Optical Fibre Factory, Hebei, China) and an inverted IX71 fluorescence microscope (Olympus, Tokyo, Japan) equipped with a 100-W mercury lamp, an excitation filter (BP 420 ± 20 nm), a dichromatic mirror (DM 455) and a fiber optic spectrometer QE65000 (Ocean Optics, Dunedin, FL, USA) attached to the side port.

### 3.2. Preparation of GSH Stabilized QDs

Briefly, oil-soluble CdSe-ZnS core-shell QDs were purchased from JIAYUAN Quantum Dots Co. Ltd. (JIAYUAN, Wuhan, China) and dissolved in chloroform to 8.0 μM. GSH stabilized QDs were synthesized based on the previously reported procedures of the exchange of TOPO on the surface of as-synthesized QDs by GSH [32]. QDs were dissolved in chloroform, to which a 100 μL GSH solution (containing 0.142 g GSH and 40 mg KOH in 2 mL methanol) was added followed by vigorous shaking. After the addition of 1.5 mL NaOH aqueous solution (1 mM), the top aqueous layer was separated and precipitated with NaCl and methanol to remove excess GSH. The resulting QDs were dissolved in 500 μL borate buffer (pH 8.5, 10 mM). The concentration of QDs was measured based on the previously reported method [33].

### 3.3. Preparation of QDs-Protein A and QDs-IgG Conjugates

The method involves EDC and NHS to form active esters to conjugate the carboxyl of QDs’ surface to amine compounds of Protein A. Specific methods: Activate the QDs by mixing 10 μL QDs (8 μM) with 2 mg EDC and 1 mg NHS in 30 μL 0.1 M borate buffer. Incubate for 30 min at room temperature with continuous gentle mixing. Combine activated QDs and 32 μL Protein A (1 mg/mL) and incubate at room temperature for one hour with continuous gentle mixing. Precipitation was romoved by centrifugation and excess QDs were removed by ultrafiltration. Then 32 μL IgG (1 mg/mL) was added and incubated for 30 min at room temperature to form QDs-IgG.

### 3.4. Procedure of Capillary Electrophoresis

CE experiments were all performed in 75 μm ID × 60 cm long fused-silica capillaries. The effective length (length from injection to the detection window) was 35 cm. When a capillary was firstly used, it was rinsed with 0.1 M HCl, pure water, 0.1 M NaOH, pure water and electrophoretic buffer sequentially for 20 min, respectively. Hydrodynamic injection was performed by siphoning at 15 cm height differences for 20 s at anode. A solution of 25 mM Na_2_B_4_O_7_ (pH 9.3) was used as CE separation buffer. Before analysis, the capillary was injected by high pressure and equilibrated with running buffer for 15 min. The separation was achieved at room temperature. Between each run, the capillary was washed with running buffer for 10 min to ensure the reproducibility.

### 3.5. Agarose Electrophoresis

Analysis of QDs-IgG conjugation was performed by electrophoresis using a Minicell Primo (Thermo, Pittsburgh, PA, USA) with 1% Omnipur agarose (EMD) in 10 mM Na_2_B_4_O_7_·10H_2_O (adjusted to pH 8.0 using 1 M HCl) at 7.9 V/cm for 15 min. Six fold loading buffer (16% sucrose in H_2_O) was added to samples before loading. For purification, the buffer was cooled on ice, the electrophoresis apparatus was surrounded in ice, and the gel was run at 6.4 V/cm for 20 min. Gels were visualized under 305 nm UV with a ChemiImager 5500 (Alpha Innotech Corporation, San Leandro, CA, USA) for analysis.

### 3.6. Preparation of QDs-Immunocomplex

After the formation of QDs-IgG, then add DyLight labeled goat anti-human IgG, and shake at room temperature with different time. The resulting solution contained stable QDs-immunocomplex without obvious aggregates was ready for assay.

## 4. Conclusions

In summary, we have systematically studied QDs and IgG conjugation and antigen-antibody interaction using CE-FL. Protein A was conjugated to QDs by coupling agents. Protein A bound QDs species were clearly separated by the CE-FL method. CE-FL provides a facile, fast, highly sensitive, relatively inexpensive and disposable device for rapid measurement of ligand-particle interaction. It indicated very good resolution and showing stable assembly of QDs-immunocomplex. The FRET process occurred between QDs and DyLight in the immunocomplex. This method can be applied to the detection of the antigen-antibody reactions, immunoassay and the interaction between biomolecules, *etc*.
